# Retention of *E*. *coli* and water on the skin after liquid contact

**DOI:** 10.1371/journal.pone.0238998

**Published:** 2020-09-17

**Authors:** Ana K. Pitol, Tamar Kohn, Timothy R. Julian

**Affiliations:** 1 Eawag, Swiss Federal Institute of Aquatic Science and Technology, Dübendorf, Switzerland; 2 Laboratory of Environmental Chemistry, School of Architecture, Civil, and Environmental Engineering (ENAC), École Polytechnique Fédérale de Lausanne (EPFL), Lausanne, Switzerland; 3 Swiss Tropical and Public Health Institute, Basel, Switzerland; 4 University of Basel, Basel, Switzerland; Qatar University, QATAR

## Abstract

The frequent contact people have with liquids containing pathogenic microorganisms provides opportunities for disease transmission. In this work, we quantified the transfer of bacteria—using *E*. *coli* as a model- from liquid to skin, estimated liquid retention on the skin after different contact activities (hand immersion, wet-cloth and wet-surface contact), and estimated liquid transfer following hand-to-mouth contacts. The results of our study show that the number of *E*. *coli* transferred to the skin per surface area (n [*E*. *coli*/cm^2^]) can be modeled using *n* = *C* (10^−3.38^+*h*), where *C* [*E*. *coli*/cm^3^] is the concentration of *E*. *coli* in the liquid, and *h* [cm] is the film thickness of the liquid retained on the skin. Findings from the *E*. *coli* transfer experiments reveal a significant difference between the transfer of *E*. *coli* from liquid to the skin and the previously reported transfer of viruses to the skin. Additionally, our results demonstrate that the time elapsed since the interaction significantly influences liquid retention, therefore modulating the risks associated with human interaction with contaminated liquids. The findings enhance our understanding of liquid-mediated disease transmission processes and provide quantitative estimates as inputs for microbial risk assessments.

## Introduction

People are frequently in contact with liquids containing microbial contaminants (bodily fluids like urine, nasal discharge, blood; environmental waters including surface waters, wastewater, and urban runoff). These contacts offer opportunities for disease transmission. To assess the risks associated with human interaction with contaminated liquids, we can use the Quantitative Microbial Risk Assessment (QMRA) framework. QMRA is a method used to estimate risks associated with exposure to pathogens and is frequently used as a decision-making tool for addressing microbial health threats [1]. For example, QMRA has been used to formulate water quality guidelines for drinking and recreational water, determine treatment recommendations for potable and non-potable water reuse schemes, or reduce risks from foodborne contamination [2–5].

One of the main components of QMRAs is the exposure assessment, which is the quantification of the microbial agent or agents to which a person is exposed through specific pathways (i.e., dietary or non-dietary ingestion, dermal exposure, inhalation). Exposure assessments are ideally aggregative, accounting for exposures from multiple transmission routes [1, 6]. An important and understudied aspect of exposure assessment in the context of activities that involve contaminated liquids is the transfer of pathogens from the liquid to the skin, followed by skin to mouth transfer. Microbial organisms present in liquids in contact with the skin result in exposure both by preferential adsorption to the skin surface as well as through remaining in liquid retained in the skin [7, 8]. As a result, the quantity of pathogens retained on the skin after liquid contact is a function of both the volume of liquid on the skin and the concentration of the agent in the liquid.

To estimate the transfer of pathogens at the skin-liquid interface QMRAs use data on the concentration of pathogen in the liquid and the amount of liquid retained on the skin. For example, Julian et al. 2018 used the volume of water retained on the skin per surface area [cm^3^/cm^2^] and the concentration of *E*. *coli* in the water [*E*. *coli*/cm^3^] to model hand contamination following water contacts. Based on the number of hand-to-mouth contacts and the transfer efficiency of pathogens from hand-to-mouth [%], exposure through non-dietary ingestion was estimated [9]. Similarly, De Man et al. 2014 used the volume of water retained on the hands and the concentration of pathogens in the water to estimate hand contamination following flooding events and used this data to calculate the risk of subsequent hand-to-mouth contacts [10].

Despite the importance of having an accurate estimate of the volume of liquid retained on the skin after hand-to-liquid contacts, the information available for water-based liquids is unreliable and incomplete [11]. The U.S. EPA Exposure Factors Handbook (2011) [12] includes estimates on the mean film thickness of liquid retained on the skin. However, unlike other exposure data provided in the Handbook, there is no information on the variation or quality of the data. Specifically, there is no information on the distribution of the data around the mean, nor on the number of replicates performed [12]. Concerningly, a follow-up study conducted five years after the original study [11] was not able to replicate the original data for water-based liquids. Nonetheless, this data, being the only available, has been used in multiple risk assessment studies [9, 10, 13]. Similarly, hand-to-mouth transfer of liquid is another important parameter in exposure assessments where data is limited. The available hand-to-mouth transfer data is uncertain and based on a small sample size (n = 4) [14].

In addition to water retention on the skin, pathogen adsorption to the skin is an important factor influencing pathogen exposures [7, 8]. The total transfer of pathogens from liquid to skin is estimated from the sum of the pathogens adsorbed on the skin and the unadsorbed pathogens present in the liquid retained on the skin. Information on the adsorption of pathogens to the skin is only available for viruses [7, 8]. In the absence of bacterial transfer data, QMRAs must rely only on virus transfer estimates [15]. Nevertheless, studies on fomite-mediated transfer of pathogens have shown a significant difference in the transfer of virus as compared to bacteria [16, 17].

In this study, we estimate *E*. *coli* transfer at the skin-liquid interface—using *E*. *coli* as a model- to provide bacteria-specific data and determine whether differences between *E*. *coli*–a Gram-negative bacteria–and virus transfer observed for fomite-mediated transfer also hold for liquid transfer. Additionally, this study presents estimates on the transfer of liquid from hand-to-mouth and estimates of water retention on the skin after water-related activities (wet-cloth contact, wet-surface contact and hand immersion) and investigates factors influencing water retention.

## Material and methods

### Ethical approvals

The protocol used in the *E*. *coli* transfer study was approved by the Research Ethics Committee of ETH Zurich, Zurich, Switzerland (2018-N-111). The liquid retention protocol was approved by the Research Ethics Committee of EPFL, Lausanne, Switzerland (HREC 026–2017). Written informed consent from the participants was obtained before the experiment.

### Transfer of *E*. *coli* from liquid to skin

#### Cultivation and enumeration of *E*. *coli*

**Fig 1:**
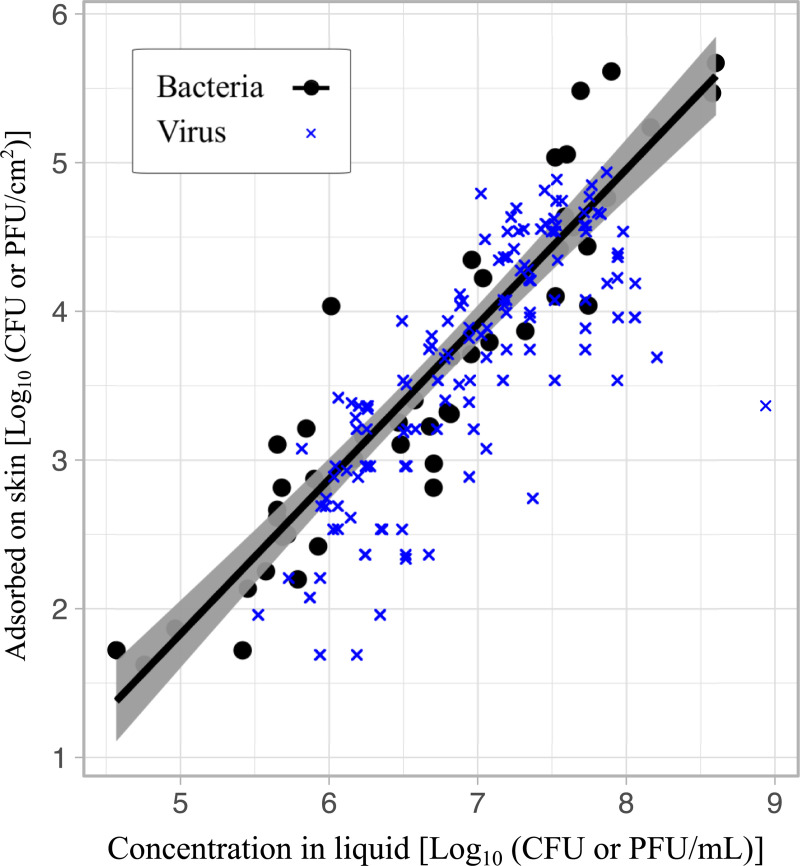
Number of bacteria and viruses adsorbed in the skin per surface area as a function of concentration. The plot shows the log_10_ transformed bacteria (*E*. *coli)* [black circles] and viruses (combined data of adenovirus, coxsackievirus, and MS2, [8]) [blue x] adsorbed on the skin per surface area as a function of the log_10_ transformed concentration of bacteria or virus in the liquid. The regression line represents the linear regression model for the number of *E*. *coli* adsorbed per surface area as a function of concentration with the 95% CI shown in grey.

*E*. *coli* DSM 11250 was used as a model organism to study the transfer of bacteria from liquid to skin. The day before the experiment, *E*. *coli* stored at -80°C were inoculated into 15 mL of Tryptone Soya Broth (AppliChem) and incubated at 37°C on an orbital shaker (180 rpm) for 8–16 hours, until the concentration of bacteria in the media was ~10^9^ CFU/mL. Before the experiment, 1 mL aliquots of the *E*. *coli* culture were centrifuged at 1150 g for 5 min and washed with phosphate-buffered saline (PBS, 137 mM NaCl, 2.7 mM KCl, 10 mM Na_2_HPO_4_, 1.8 mM KH_2_PO_4_, pH 7.4) solution three consecutive times. Subsequently, the aliquots of *E*. *coli* were diluted to one of the desired concentrations between 10^5^–10^8^ CFU/mL (see Fig 1). The spread plate technique was used to enumerate *E*. *coli*. Briefly, samples were serially diluted on PBS, plated (100μL of sample per plate) on TBX Agar (Sigma-Aldrich), incubated overnight at 37°C, and enumerated the following day.

#### *E*. *coli* transfer trials

The transfer of *E*. *coli* at the skin-liquid interface was studied in a trial conducted by nine volunteers. The experiments were performed using the finger pads of the volunteers since it is expected that most hand-to-mouth contacts are to be performed using the fingers and previous work on pathogenic viruses and bacteriophage transfer from liquid to skin showed no difference in the transfer of viruses in different parts of the hands and arms [8]. The method used herein is analogous to the methods used elsewhere to quantify the transfer of virus and bacteria from fomites to fingers [16–19]. Three fingers of each hand were selected for experimental samples and one for negative control, for a total of six transfer events and one negative control per volunteer. The experimental method used to quantify the transfer of *E*. *coli* from liquid to the skin is an adaptation of the method described elsewhere for virus transfer [7]. In brief, a circular area (diameter = 5mm) was delimited on the hand of the volunteers using the rim of a 20 μL pipette tip coated in Vaseline (Vifor Pharma, Switzerland). Subsequently, 20 μL of *E*. *coli* solution at a concentration of between 10^5^ and 10^8^ CFU/mL was added to the area inside the Vaseline and removed after five seconds using a pipetman. The area inside the Vaseline was then sampled two consecutive times: the first sampling was performed by pipetting up and down once using PBS; this was done to recover the *E*. *coli* present in the liquid that was retained on the skin (the unadsorbed *E*. *coli*). The second sample recovered the *E*. *coli* adsorbed on the skin and was performed by pipetting up and down five times using beef extract solution (3% beef extract (Sigma-Aldrich) - 0.1 M glycine (Fluka)- pH 8). Beef extract was used to desorb the adsorbed bacteria from the skin, in consistency with the previously developed protocol for virus transfer [7, 8].

One negative control (PBS), one positive control (PBS + *E*. *coli*), and one growth control (PBS + *E*. *coli* + beef extract) were processed alongside each experiment. The growth control was used to determine if the concentration of *E*. *coli* in the recovered sample was influenced by the beef extract. To minimize changes in the concentration of *E*. *coli* over time (due to inactivation or growth); all the samples were enumerated within 1 hour of the transfer experiment. Additionally, the samples were kept on ice during the experiment and until enumeration.

### Liquid retention on the skin

#### Survey of skin characteristics

A survey was conducted to investigate factors that could potentially influence the retention of liquid on the skin. The survey was carried out before the experimental trials and included questions directed to the participants about their age, gender, and whether they washed their hands, used alcohol-based hand sanitizer, or applied skin products within two hours prior to the experiment (S1 File).

#### Liquid film thickness

The film thickness of the liquid retained on the skin per surface area, *h* [cm], was estimated as:

$$h=\frac{V}{{SA}_{i}}$$ (1)

where *V* [mL, equivalent to cm^3^] is the volume of liquid retained on the skin, and *SA*_*i*_ [cm^2^] is the skin surface area in contact with the liquid for activity *i*.

#### Hand surface area

The surface area of the volunteers palm and whole hand were estimated using a method described elsewhere [20], based on the relationship between two-dimensional hand tracing and hand surface area [21]. Briefly, the palm of the volunteers was traced onto paper and the perimeter and area of the traced hand was quantified using an image processing package (Fiji, Version 1.0). Additionally, the thickness of the hand was estimated by measuring the second knuckle of the middle finger with a calliper. Total hand surface area was estimated as:

$${SA}_{total}=2\;{SA}_{palm}+{SA}_{perim}$$ (2)

$${SA}_{perim}=L_{perim}\;H$$ (3)

where *SA*_*total*_ is the total hand surface area [cm^2^], *SA*_*palm*_ is the surface area of the palm [cm^2^], and *SA*_*perim*_ [cm^2^] is the surface area of the perimeter calculated using Eq 3, where *L*_*perim*_ [cm] is the perimeter length and *H* [cm] is the height of the hand -assumed to be the same across the whole hand as the height of the second knuckle of the middle finger [20].

#### Quantification of volume of liquid retained on the skin

To quantify the volume of liquid retained on the skin after contact, two methods were employed: one using a “tracer”, and the other using “weight differential”. The tracer method was used in all of the liquid-contact experiments, and the weight differential method was used in a subset of experiments to validate the tracer method, due to the inability to accurately weigh liquids transferred during the experiments on hand-to-surface transfer and hand-to-mouth transfer (see S2 File). The weight differential method consisted of weighing the liquid before and after the activity was performed and assuming the difference was equal to the mass of liquid retained on the skin. The measured mass was converted to an estimated volume by dividing the mass (*m* [g]) by the density (δ [g/cm^3^]) of the liquid. When water was used, the density was assumed to be 1.00 g/cm^3^. When beet root juice was used (for the tracer method described below), the density used was 1.03 ± 0.01 g/cm^3^, which was estimated empirically at 25°C by dividing the mass of the liquid by its volume. The mass was measured by pipetting 30 times a volume of 2 μL of beet root juice and weighing it using the Excellence Plus Balance (Mettler Toledo, Switzerland).

The tracer method used to quantify the volume of liquid retained on the skin is a variation of the colorimetric quantification method described elsewhere [22, 23]. Beet root juice (food quality, 100% beet root juice, Biotta AG, Switzerland) was used as tracer liquid because of the sufficiently high concentrations of betalains (naturally occurring red pigments suitable for quantification using spectrophotometric techniques) and volunteer safety. After performing the activities (described below), the volunteer's hand was introduced into a sampling bag (Whirl-pak bag, NASCO Corp., USA) containing either 200 or 300 mL of Nanopure water and massaged for 10 seconds to remove the beet root juice retained on the skin. Two samples of 1.0 mL were collected from the sampling bag for subsequent analysis. The samples were analyzed spectrophotometrically at a wavelength of 530 nm (Biochrom Libra S4) and the volume of liquid retained (*V* [cm^3^]) was calculated as follows:

$$V=\frac{V_{s}A_{s}}{A_{orig}-A_{s}}C_{f}$$ (4)

where *A*_*s*_ and *A*_*orig*_ are the absorbances (Absorbance Units, or AU) of the sample and of the original beet root juice, *V*_*s*_ is the volume of liquid in the sampling bag used to rinse the hand of the volunteer (mL), and *C*_*f*_ is a correction factor (unitless) to adjust estimates of liquid retention based on the tracer method to align with estimates obtained using the weight differential method. *C*_*f*_ is estimated using data obtained in experiments in which both the tracer method and weight differential method were used. More details on the spectrophotometry analysis and the validation of the tracer method can be found in the supporting information (S2 File).

#### Liquid-to-hand transfer trials

Liquid-to-hand transfer trials were conducted by 40 adult volunteers to mimic three different activities involving hand-to-liquid contacts: 1) hand contacting a wet cloth, 2) hand contacting a wet surface, and 3) full hand immersion into the liquid. Each volunteer was asked to perform one of the three different activities per experiment, with each volunteer performing between one and three experiments.

*Wet-cloth contact*. The volunteers were asked to use the palm in two different activities: wring a cloth saturated with beet root juice for five seconds or press the hand on the cloth saturated with beet root juice for five seconds. The selection of the order in which the volunteer performed the activity (wringing or pressing) and the hand (left or right) was randomized. After performing one activity, the volunteers were asked to wash and dry their hands and subsequently perform the next activity. Two different textile materials were used, with one material per hand: 100% polyester (MIGROS Cocina & Travola, Turkey) and 100% cotton (MIGROS Cocina & Travola, India). Immediately after each contact event, the volunteer's hand was introduced into a sampling bag containing 200 mL of Nanopure water and the hand was massaged for 10 seconds. Three samples of 1 mL were collected from the sampling bags for spectrophotometric analysis together with a sample of the original beet root juice.

*Wet-surface contact*. The volunteers were asked to press the palm on a wet surface (either polyvinyl chloride (PVC) plastic or stainless steel, (length = 23cm, width = 23cm)) for five seconds. Before the experiment, the surfaces were inoculated with five milliliters of beet root juice, which was subsequently distributed throughout the entire area with an L-shaped cell spreader (VWR). After each contact event, the hands were sampled as described above and three samples of 1mL were used for spectrophotometric analysis. The activity was performed before and after handwashing with soap.

*Hand immersion*. The volunteers were asked to dip their hand in a glass containing 500mL of beet-juice for 5 seconds. To estimate the liquid retention at time zero after contact, the volunteers removed the hand and introduced it immediately into a sampling bag containing either 200mL or 300mL of Nanopure water. To estimate the liquid retention ten seconds after contact, the volunteers removed the hand and placed it above the container for ten seconds before introducing it into the sampling bag. Three samples of 1mL were collected for spectrophotometric analysis together with a sample of the original beet root juice taken from the glass for calibration.

To validate the volume quantification method, a subset of the immersion experiments were performed using water with one hand and beet root juice with the other hand. When water was used, the retained volume was estimated using the weight-difference method. When beet root juice was used, the retained volume was estimated using both the tracer and weight differential methods.

#### Hand-to-mouth transfer trials

To estimate the liquid retained on the mouth after hand-to-mouth contacts, the volunteers were asked to press their fingertip on a plastic weighing boat containing 1 mL of beet root juice and subsequently contact the mouth with the fingertip. The beet root juice retained on the finger was recovered by pressing the finger on a plastic weighing boat containing 1 mL of nano-pure water for 5 seconds. The experiment was repeated four times, using four fingers from one hand. As a control, the volunteers were asked to repeat the experiment with the other hand. In the control samples, the beet root juice retained on the finger was recovered without performing hand-to-mouth contact. The percentage of liquid transferred (T[%]) was defined as follows:

$$T=\left(1-\frac{A_{h-m}}{A_{c}}\right)\;100\%$$ (5)

where *A*_*h*−*m*_ is the absorbance of the liquid recovered after hand-to-mouth contact [AU] and *A*_*c*_ is the absorbance of the liquid recovered from the corresponding control finger [AU].

### Statistical analyses

All statistical analyses were performed using R statistical software (The R Foundation for Statistical Computing Platform, version 3.4.4). Statistical significance was defined using α < 0.05.

## Results

### *E*. *coli* transfer model

A total of 54 liquid to skin transfer events were performed with nine volunteers using *E*. *coli* concentrations between 10^5^−10^8^ CFU/mL. Five out of the 54 (9%) data points were unusable because the *E*. *coli* on the agar plates were unable to be counted for at least one of the three samples (*E*. *coli* added, unadsorbed *E*. *coli* recovered, and adsorbed *E*. *coli* recovered). In addition to the samples, one negative control, one positive control, and one growth control were processed alongside each experiment. None of the negative controls showed *E*. *coli* contamination. Additionally, there was no statistically significant difference between the positive control and the growth control (paired t-test; t (7) = 2.19, p = 0.06), which implies that there was no significant impact of the beef extract solution on *E*. *coli* estimates.

As observed in Fig 1, *E*. *coli* adsorption to the skin scaled linearly to the concentration of bacteria in the liquid (F(1,46) = 312, p<0.001, R^2^ of 0.87). The number of *E*. *coli* adsorbed on the skin can be described by the following model:

$$n_{ads}=10^{b}\;C^{m}$$ (6)

where *n*_*ads*_ [*E*. *coli*/cm^2^] is the number of *E*. *coli* adsorbed in the skin per surface area, C [*E*. *coli*/mL] is the concentration of *E*. *coli* in the liquid, with *b* = -3.38 ± 0.39 (-2.58 - -4.17) and *m* = 1.04 ± 0.06 (1.16–0.92) (mean ± SE (95% Confidence Interval)) as empirically derived parameters. It is worth noting that *m* is not significantly different from unity (one), therefore, Eq 6 can be reduced to the linear form *n*_*ads*_ = 10^*b*^ *C*.

To determine if the number of *E*. *coli* retained on the skin is significantly different than the number of virus retained on the skin, we compared the adsorption of *E*. *coli* from this study with our prior data on the adsorption of adenovirus, coxsackievirus and bacteriophage MS2 [8] (Fig 1). Multiple linear regression was used to predict the number of pathogens adsorbed to the skin as a function of concentration and pathogen type: bacteria (*E*. *coli*) vs virus (combined data of adenovirus, coxsackievirus and MS2, data from [8]) (F(2,185) = 185, p<0.001, R^2^ = 0.87). The model revealed a small but significant difference between *E*. *coli* and the combined data of the adsorption of adenovirus, coxsackievirus and MS2 to the skin (β = -0.20, SE_ β = 0.09, p = 0.026). The difference between the adsorption of *E*. *coli* and viruses is dependent on the concentration. For example, the amount of viruses adsorbed on the skin is on average 0.35 log_10_ higher than the amount of *E*. *coli* adsorbed on the skin when the concentration of *E*. *coli* or viruses in the liquid is 10^5^ per mL. In contrast, the amount of viruses adsorbed to the skin is 0.05 log_10_ higher than *E*. *coli* when the concentration is 10^7^ *E*. *coli* or viruses per mL.

Prior work on liquid-mediated transfer of virus described pathogen transfer as the addition of the number of pathogens present in the liquid retained on the skin (the unadsorbed fraction, *n*_*unads*_) and the pathogens adsorbed on the skin (*n*_*ads*_) [7]. Consequently, the total transfer of *E*. *coli* from liquid to skin can be estimated using the following equation:

$$n=n_{ads}+n_{unads}$$ (7)

or

$$n=10^{-3.38}\;C^{1.04}+Ch$$ (8)

where *n* [*E*. *coli*/cm^2^] is the number of *E*. *coli* transferred to the skin per surface area, *C* [*E*. *coli*/mL] is the concentration of *E*. *coli* in the liquid, *h* [cm] is the thickness of the liquid retained on the skin after the liquid-contact activity. Since *m* is not significantly different than one, Eq 8 can be reduced to:

$$n=C\;(10^{-3.38}+h)$$ (9)

### Estimates of liquid film thickness (h)

#### Hand immersion

Hand immersion experiments were performed with 30 volunteers in two trials. In the first trial, sampling was carried out immediately after hand immersion, in the second trial sampling was performed ten seconds after hand immersion, allowing part of the liquid to fall back into the container before sampling. Retaining the hand for ten seconds above the flask before sampling significantly reduced the film thickness of the liquid on the skin (t-test, t (37) = -7, p = <0.001). Allowing the liquid to fall back into the container for ten seconds reduced the liquid film thickness by 49% (Table 1).

**Table 1:** Thickness of liquid retained on the skin after liquid contact.

activity	sampling time^a^ [sec]	film thickness [cm] mean ± SD	subjects	n
Hand immersion	0	0.0078 **±** 0.0029	15	30
Hand immersion	10	0.0038 **±** 0.0011	30	30
Wet-cloth contact	10	0.0039 **±** 0.0016	19	76
wringing^b^		0.0035 **±** 0.0013		38
pressing^b^		0.0044 **±** 0.0016		38
wet-surface contact	10	0.0046 **±** 0.0016	15	60
metal		0.0041 **±** 0.0015		30
plastic		0.0050 **±** 0.0017		30

^a^ Amount of time spent after the activity and before the sampling of the hand.

^b^ The data for cotton and polyester were combined, as there was no statistically significant difference in film thickness between both materials (ANOVA, F(1,73) = 0, p = 0.99).

#### Wet-cloth contact

A total of 76 wet-cloth contact events, which consisted of wringing or pressing a cloth (polyester or cotton) saturated with liquid were carried out with 19 volunteers (Table 1). Pressing the cloth transferred on average 22% more liquid to the skin than wringing the cloth, a statistically significant difference in film thickness (ANOVA, F (1, 73) = 5.45. p = 0.022). In contrast, no significant difference on film thickness was found when cotton was used as compared with polyester (ANOVA, F(1, 73) = 0, p = 0.996) (Table 1).

#### Wet-surface contact

A total of 60 wet-surface contact events, which consisted of pressing the hand on a surface saturated with liquid, were carried out with 15 volunteers using two different surface materials: metal and plastic. Each volunteer touched both surfaces, one surface with each hand. The film-thickness retained on the skin was significantly influenced by the surface material (paired samples t-test, t (59) = -4.8, p<0.001). Specifically, the transfer of liquid to the skin was 20% higher when the contact material was plastic as compared with metal (Table 1).

#### Factors influencing liquid retention

One-way ANOVA test revealed that the activity performed (wet-cloth contact, wet-surface contact and hand immersion) had a small but significant influence on the liquid film thickness (ANOVA, F (2, 163) = 3.45, p = 0.034). Tukey's post-hoc comparisons showed a borderline-significant difference in liquid retention when participants performed wet-cloth contact as compared with wet-surface contact (p = 0.05). Liquid film thickness after hand immersion was not significantly different than after wet-cloth contact (p = 0.94) or wet-surface contact (p = 0.09) (Table 1).

Multiple linear regression was used to estimate if hand characteristics, as defined by the survey, could potentially explain some of the variation observed in the liquid film thickness. The factors analysed included humidity and temperature, participant's gender, and whether the participant performed any of the following activities before conducting the experiment: hand-washing, application of ethanol-based disinfectant, application of topical products such as moisturizer. None of the tested factors significantly influenced liquid retention on the skin (Multiple Regression, F(9,40) = 1.01, R^2^ = 0.18, p = 0.451).

### Hand-to-mouth liquid transfer

The transfer of liquid from hand-to-mouth was estimated as the percentage of the total volume that was transferred. A total of 44 transfer events were carried out with 11 volunteers. The percentage of the liquid on the finger that was transferred to the mouth after contact was 50 ± 19% with transfers ranging from 8 to 78% (min-max).

## Discussion

This work presents a model to estimate the number of *E*. *coli* transferred to the skin per surface area as a function of the concentration of *E*. *coli* in the liquid that accounts for both, the bacteria attached to the skin (adsorbed) and the bacteria present in the liquid retained on the skin (unadsorbed). Findings from the *E*. *coli* transfer experiments show a significant difference between the adsorption of *E*. *coli* to the skin and the adsorption of viruses (combined data of adenovirus, coxsackievirus, and MS2) to the skin. Our results are consistent with previous studies, which show that the transfer of viruses between hands and fomites is significantly different than the transfer of Gram-negative bacteria between hands and fomites [16, 17]. The estimates for transfer of *E*. *coli* at the liquid-skin interface can be used to improve estimates of indirect transmission involving liquid-hand contacts [15] and are complementary to the previous findings of virus transfer at the skin-liquid interface [7, 8].

This work also presents estimates for liquid retention on the skin after skin-liquid contact events, which are necessary to perform microbial risk assessments of water related activities that could lead to non-dietary ingestion of pathogens. Findings from the liquid retention experiments demonstrate that time elapsed post-activity is the single most important factor influencing liquid retention, which strongly influences subsequent associated risk. For example, in activities such as children playing in water, we would expect frequent hand-to-liquid and hand-to-mouth contacts -therefore, less time elapsed post-activity and more water retained on the hand- as compared to activities such as fishing where we would expect less contact frequency. Our model shows that, if a person immerses the hand in water containing 10^3^ *E*. *coli*/mL, immediately after the immersion the hand will have 8.2 *E*. *coli*/cm^2^, which is equal to *C* (10^−3.38^+*h*), where *C* = 10^3^ *E*. *coli*/mL, and *h* = 0.0078 cm. After ten seconds, we observe a ~50% reduction in the liquid film on the skin (*h* = 0.0038 cm), which reduces *E*. *coli* retained on hand to an estimated 4.2 *E*. *coli*/cm^2^. The reduction in *E*. *coli* is caused by the unadsorbed fraction of *E*. *coli* that is removed from the skin with the liquid dripping off the skin. Conversely, the activity performed had only a small influence on the transfer of bacteria from liquid to skin. If a person performs hand immersion, has contact with a wet cloth or touches a surface containing water with 10^3^ *E*. *coli*/mL, ten seconds after performing the activity the contamination on the hand would be 4.2, 4.3, and 5 *E*. *coli*/cm^2^ for hand immersion, wet-cloth contact, and wet-surface contact respectively.

Additionally, skin characteristics did not influence water retention on skin. Prior research has demonstrated that exogenous factors such as temperature, humidity or the use of topical products have an impact on the hydration of the skin [24], and skin hydration could potentially influence water retention. However, we found no correlation between the use of topical products, participant gender, temperature, and humidity on the retained liquid film thickness. This does not imply that the factors analyzed do not influence liquid transfer. However, any impact is insufficient to be observed with the relatively small sample size (less than 40 participants) and/or does not act within the restricted range of factor values tested. For example, humidity and temperature were very similar for all trials since most of the trials were performed in the same room. Failing to observe an influence of those factors on liquid transfer may suggest that their relative contribution on liquid transfer is small. These factors, therefore, appear to not be relevant for risk assessments, however future studies with larger sample sizes may be needed.

In addition to the transfer of contaminated liquid to the hands, the transfer of contaminated liquid from the hands to the mouth is another important parameter in microbial risk assessments where available data is limited. Here, we estimate approximately 50 ± 19% of liquid is transferred on finger-to-lip contact. This value is similar to the findings of Gorman et al. 2014 [14]. In their study of transfer of the oil, vinegar, and powder, 36.8 ± 31.9% of vinegar was transferred from the finger to the mouth as determined using a chemical tracer method. The difference observed can be attributed to a difference in the experimental method and in the sample size. Their study had a small sample size (n = 4), and there was a high uncertainty associated with the chemical analysis of the tracer substance present in the recovered saliva. Conversely, our study had a higher sample size (n = 44) and a more reliable quantification method.

Our research has a number of limitations. Findings from *E*. *coli* adsorption may not be generalizable to other bacteria: different bacteria may have different transfer rates as observed for fomite-mediated transfer [25]. Given the model of liquid-mediated transfer that includes both adsorbed and unadsorbed fractions, the mechanism driving this difference would manifest in the adsorbed fraction, where adsorbed fractions would vary by bacterial species or strain. The findings of this study are restricted to *E*. *coli*. Differences in transfer efficiencies between fomites and skin have been observed between Gram-positive and Gram-negative bacteria [16, 17]. Therefore, further work needs to be done to establish whether bacterial transfer at the skin-liquid interface is influenced by the species or type of bacteria, including cell wall composition (Gram negative vs. Gram positive). Additionally, *E*. *coli* concentrations between 10^5^–10^8^ CFU/mL were used in the transfer experiments. Although concentrations lower than 10^5^ CFU/mL are relevant for most exposure assessments, testing the transfer of *E*. *coli* at lower concentrations resulted in *E*. *coli* counts close to or below our limit of detection (10 CFU/cm^2^). Furthermore, there was a significant difference between the two methods used to quantify liquid retention. Therefore, a correction factor was used to adjust the tracer method such that it aligned with the weight-differential method. Development of higher resolution methods for capturing liquid transfer at low volumes will help to improve the accuracy and precision of our estimates.

Despite the limitations, the estimates presented in this study can help to reduce uncertainty and improve the accuracy of dermal and non-dietary exposure assessments for human interaction with contaminated liquids. In this work, we present quantitative estimates for the transfer of bacteria–using *E*. *coli* as a model–at the skin-liquid interface. Estimates of pathogen transfer between liquids and skin were only available for viruses and are necessary to quantify indirect transfer of pathogens. Additionally, this article provides estimates on liquid retention on hands and mouth, which are necessary to develop microbial risk assessments and were currently incomplete and poorly characterized.

## Supporting information

S1 File Skin characteristics survey. (DOCX)

S2 File Quantification of volume of liquid retained on the skin. (DOCX)
