# Supplementary material for: Retention of E. coli and water on the skin after liquid contact
Source: PLoS One. 2020 Sep 17;15(9):e0238998. doi: 10.1371/journal.pone.0238998 (PMC7498081; doi:10.1371/journal.pone.0238998)
Supplement: S1 File — (DOCX) [file pone.0238998.s001.docx]

**Article title:** Retention of *E. coli* and water on the skin after liquid contact

**Authors:** Ana K. Pitol, Tamar Kohn, Timothy R. Julian

**File 2. Skin characteristics survey**

Date:

Time:

Temperature (°C):

Humidity:

1. Have you washed your hands in the last 2 hours, including showering? [Yes, No]

2. Have you used any alcohol-based disinfectant in the last 2 hours? [Yes, No]

3. Have you applied skincare products (moisturizer) in the last 5 hours? [Yes, No]

4. If ANSWER to 3 is YES, describe product category: [open question]

5. How old are you? [2-5], [6-12], [13-18], [19-25], [26-35], [36-45], [>45]

6. What's your sex at birth? [Female, Male]
