# Supplementary material for: Retention of E. coli and water on the skin after liquid contact
Source: PLoS One. 2020 Sep 17;15(9):e0238998. doi: 10.1371/journal.pone.0238998 (PMC7498081; doi:10.1371/journal.pone.0238998)
Supplement: S2 File — (DOCX) [file pone.0238998.s002.docx]

**Article title:** Retention of *E. coli* and water on the skin after liquid contact

**Authors:** Ana K. Pitol, Tamar Kohn, Timothy R. Julian

**File 4. Quantification of volume of liquid retained on the skin**

This section of the Supporting Information describes the development and validation of the method used to quantify the volume of liquid retained on the skin (the “tracer method”).

**Text 4.1. Relationship between absorbance and the concentration of beet root juice**

To test if there is a linear relationship between the concentration of beet root juice (food quality, 100% beet juice, Biotta AG, Switzerland) and the absorbance (A [AU]) measurements, six different calibration curves were performed using six batches of beet root juice. Serial dilutions (1:2) were performed using the original beet root juice. Subsequently, the absorbance of each sample in the dilution series was measured in duplicate with the spectrophotometer (Biochrom Libra S4) at a wavelength of 530 nm. Finally, the theoretical absorbance output was compared with the measured output.

**Results:** There is a linear relationship between the concentration of beet root juice and the absorbance measurements (linear regression, F (1,46) = 10^5^, p< 0.001, R^2^ > 0.99).

**Text 4.2. Estimating volume based on absorbance measurements: “the tracer method”**

After performing the liquid contact activities (described in the paper), the volunteer's hand was introduced into a sampling bag containing either 200 or 300 mL of Nanopure water ($V_{s}$ [mL, equivalent to cm^3^]), depending on the activity. Two samples of 1 mL were collected from the sampling bag and analyzed spectrophotometrically. The volume of liquid retained on the skin ($V$ [cm^3^]) was calculated with the following equation:

$V= \frac{V_{s} A_{s}}{{(A}_{orig} - A_{s})}$ (2)

where $A_{s}$ and $A_{orig}$ are the absorbances [AU] of the sample and of the original beet root juice and $V_{s}$ is the volume of liquid in the sampling bag used to rinse the hand of the volunteer [mL].

**Text 4.3. Comparing the tracer method with weight differential method**

The tracer method was used in all of the liquid-contact experiments, and the weight differential method was used in a subset of experiments to validate the tracer method. The weight differential method was performed by weighting the liquid before and after the activity using the Excellence Plus Balance (Mettler Toledo, Switzerland). The mass of liquid retained on the skin [g] was assumed to be the same as the difference in the mass of the liquid before and after the activity. The volume of liquid retained on the skin was estimated by dividing the mass [g] by the density (𝛿 [g/cm^3^]) of the liquid. A total of 60 hand immersion events were carried out with 30 volunteers. Each volunteer performed the experiment with water using one hand and with beet root juice using the other. Whenever the experiment was performed using beet root juice, the quantification of volume of liquid retained on the skin was carried out using both, the weight differential method and the tracer method.

**Results:** There was a statistically significant influence of the quantification method on the estimated volume of liquid retained (ANOVA, F (84, 2) = 19.55, p <0.001). Tukey post hoc test showed a statistically significant difference in the liquid volume estimated using the tracer method as compared with the weight differential method for both water and beet juice (Tukey HSD, p < 0:001; p < 0:001). When using beet root juice, the weight-difference method estimated a volume that was 37% higher than the one estimated using the tracer method. In contrast, there was no statistically significant difference for the weight-difference method used for hand immersion in water as compared to hand immersion in beet juice (Tukey HSD, p = 0.88).

**Text 4.4. Correction factor**

Based on the difference in the volume of liquid retained on the skin after hand immersion estimated using the weight differential method ($V_{weight})$as compared with the tracer method ($V_{A})$, a correction factor ($C_{f}$ ) was calculated as follows:

$C_{f}= \frac{V_{weight}}{V_{A}}= \frac{1.779}{1.224}=1.45$ (3)

The $C_{f}$ was used to correct all the measurements of liquid transfer calculated using the spectrophotometric method.

**Text 4.5. Method validation**

Additional experiments were performed to validate the correction factor applied to the tracer method. A subset of the samples (n=76) obtained in the wet-cloth contact experiment (see paper), performed with beet root juice, were quantified using both methods, the tracer method and the weight differential method. The volume of liquid retained on the skin estimated using the tracer method was calculated using the corrected equation:

$V_{A} C_{f} =V_{weight}$ (4)

**Results:** Using the corrected equation, there was no statistically significant difference in the volume of liquid estimated using the tracer method and the weight differential method (paired samples t-test, t (70)=0.57, p= 0.57).
